# Supplementary material for: Predictive effects of diabetes-related risk factors for falls in community-dwelling people with diabetic peripheral neuropathy based on a logistic regression model
Source: PLoS One. 2026 Jan 2;21(1):e0340262. doi: 10.1371/journal.pone.0340262 (PMC12758703; doi:10.1371/journal.pone.0340262)
Supplement: S1 File — (DOCX) [file pone.0340262.s001.docx]

# S1_File. Equations 1 and 2 generate by the logistic regression.

The variables contained in the final model were used to generate the following logistic regression:

$Z=a+b_{1}X_{1}+b_{2}X_{2}+b_{3}X_{3}+\ldots,$ (S1)

where *Z* is the natural logarithm of the odds (logit), *a* is the constant of the regression and *b* corresponds to the regression coefficients of each variable.

Then, the predicted variable of falls can be estimated as:

$Y=\frac{e^{Z}}{1+e^{Z}}$ (S2)

where *Y* is the probability of recurrent falls and *e* is the base of the natural logarithm. The probability values range from 0 to 1, with a value closer to 1 and above 0.5 predicting that the individual is likely to be a faller and a value closer to 0 and below 0.5 predicting that the individual is less likely to be a faller.

The regression coefficient β and the corresponding OR with 95% confidence intervals were calculated. A negative β and an OR lower than one indicates that the risk of falls increases if the variable decreases, while a positive β associated to an OR greater than one indicates that the risk of falls increases if the variable increases.
