# Supplementary material for: Predictive effects of diabetes-related risk factors for falls in community-dwelling people with diabetic peripheral neuropathy based on a logistic regression model
Source: PLoS One. 2026 Jan 2;21(1):e0340262. doi: 10.1371/journal.pone.0340262 (PMC12758703; doi:10.1371/journal.pone.0340262)
Supplement: S1 Table — (DOCX) [file pone.0340262.s002.docx]

# S1 Table

| **S1 Table.** **Simple logistic regression analyses for potential predictors of falls.** | | |
| --- | --- | --- |
|  | OR (95% CI) | *P* |
| Age | 1.04 (1.01-1.07) | 0.019* |
| Sex | 0.43 (0.23-0.79) | 0.007* |
| Fasting glucose level (mg/dL) | 1.00 (0.99-1.00) | 0.739 |
| Visual deficiency (%) | 0.04 (1.05-3.84) | 0.035* |
| Use of gait aid (%) | 0.88 (0.28-2.74) | 0.825 |
| Polypharmacy (%) | 0.50 (0.67-2.27) | 0.496 |
| MNSI total score | 1.23 (1.10-1.38) | <0.001* |
| Tactile sensitivity (# insensitive plantar areas) | 1.16 (1.00-1.33) | 0.048* |
| Vibration sensitivity (% of absent sensation) | 2.26 (0.81-6.33) | 0.122* |
| DPN severity (Fuzzy score) | 1.22 (1.08-1.39) | 0.002* |
| Self-reported balance problems | 3.53 (1.83-6.81) | <0.001* |
| Self-reported limbs weakness | 2.18 (1.15-4.12) | 0.017* |
| Self-reported fear of falling | 2.89 (1.54-5.43) | 0.001* |
| High risk of falling (FRT<15cm) | 2.42 (1.06-5.56) | 0,037* |

MNSI: Michigan Neuropathy Screening Instrument, FRT = functional reach test, OR = odds ratio; CI = confidence interval
