# Supplementary material for: Predictive effects of diabetes-related risk factors for falls in community-dwelling people with diabetic peripheral neuropathy based on a logistic regression model
Source: PLoS One. 2026 Jan 2;21(1):e0340262. doi: 10.1371/journal.pone.0340262 (PMC12758703; doi:10.1371/journal.pone.0340262)
Supplement: S2 Table — (DOCX) [file pone.0340262.s003.docx]

# S2_Table

**S2 Table. Individual data for Model 1 prediction including all risk factors, except DPN severity.**

**
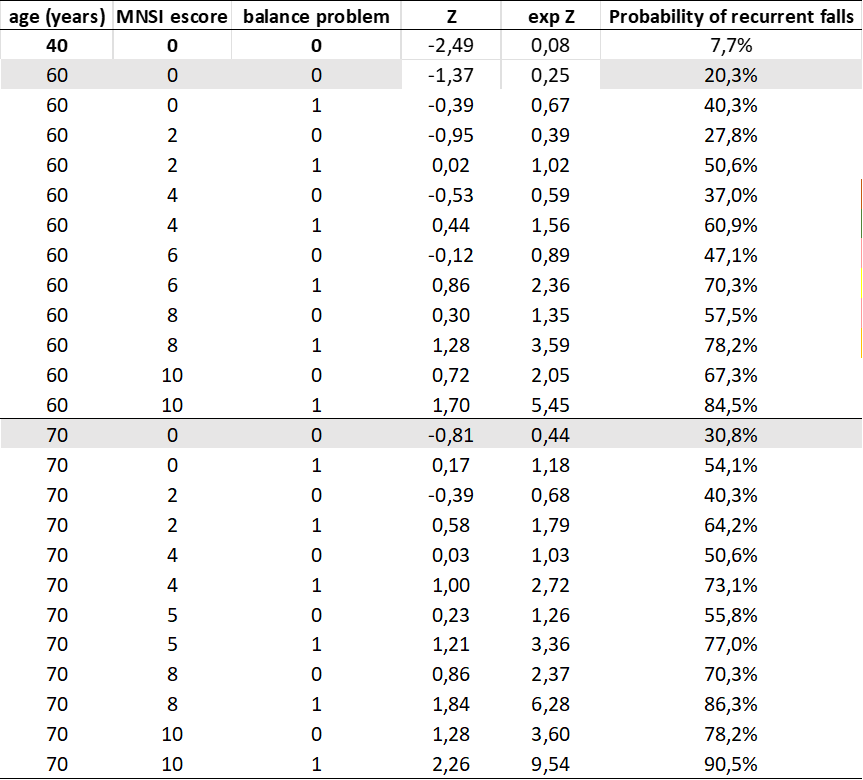
**
