# Supplementary material for: Predictive effects of diabetes-related risk factors for falls in community-dwelling people with diabetic peripheral neuropathy based on a logistic regression model
Source: PLoS One. 2026 Jan 2;21(1):e0340262. doi: 10.1371/journal.pone.0340262 (PMC12758703; doi:10.1371/journal.pone.0340262)
Supplement: S3 Table — (DOCX) [file pone.0340262.s004.docx]

# S3_Table

**S3 Table. Individual data for Model 2A prediction including all risk factors, except fuzzy system variables (MNSI score, vibration, and tactile sensitivities).**

**
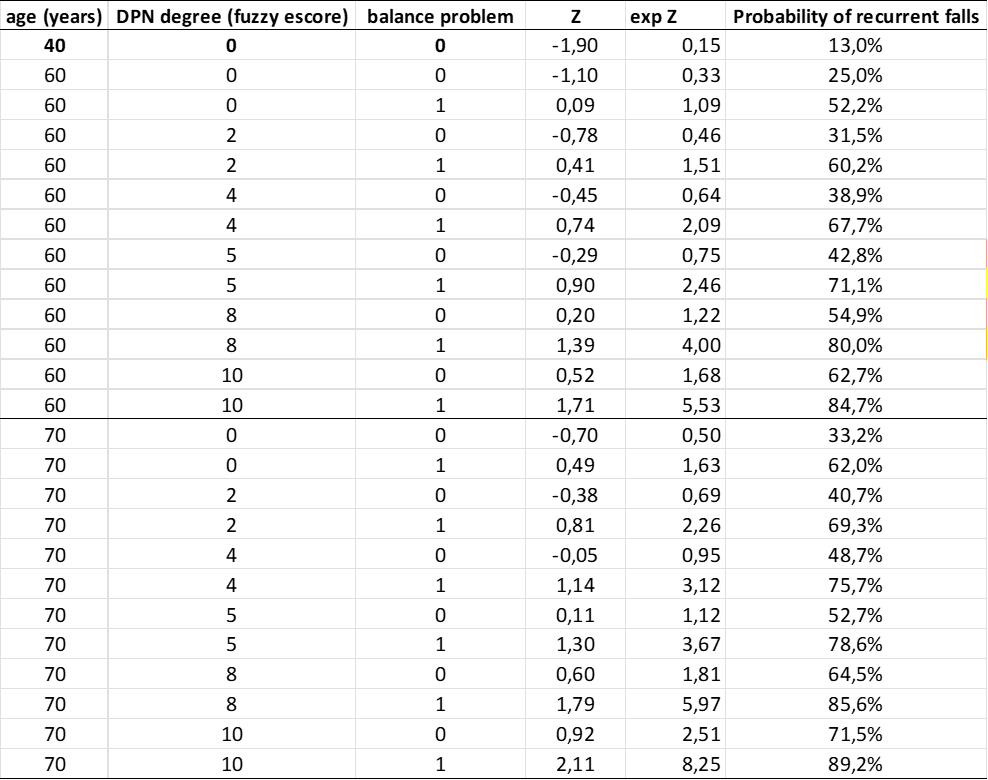
**
