# Supplementary material for: Predictive effects of diabetes-related risk factors for falls in community-dwelling people with diabetic peripheral neuropathy based on a logistic regression model
Source: PLoS One. 2026 Jan 2;21(1):e0340262. doi: 10.1371/journal.pone.0340262 (PMC12758703; doi:10.1371/journal.pone.0340262)
Supplement: Attachment — Submitted filename: Response to reviewers.docx [file pone.0340262.s006.docx]

**Response to reviewers**

São Paulo, November 4^th^ 2025

**Dear Dr Fredirick Lazaro Mashili,
Academic Editor
PLOS ONE**

We would like to thank for the opportunity of submitting a revised version of the manuscript “Interplay between diabetes-related risk factors for falls in community-dwelling people with diabetic peripheral neuropathy based on a logistic regression model” (PONE-D-24-41153). This document contains the responses to all comments raised by the academic editor and reviewers on a point-by-point basis. We hope our task fulfils the reviewers’ and editors’ expectations. Thank you once again for the opportunity, and please do not hesitate to contact us if you have any further queries and requests.

Yours sincerely. On behalf of all authors,

**PhD. Professor Isabel de Camargo Neves Sacco**

Associate Professor - MS5 III

Head of the Laboratory of Biomechanics of Human Movement and Posture

Physical Therapy, Speech and Occupational Therapy department – School of Medicine – University of São Paulo

**RESPONSES TO THE ACADEMIC EDITOR AND REVIEWERS' COMMENTS ON A POINT-BY-POINT BASIS**

**Journal Requirements:**

**When submitting your revision, we need you to address these additional requirements.**

**1. Please ensure that your manuscript meets PLOS ONE's style requirements, including those for file naming. The PLOS ONE style templates can be found at**

[**https://journals.plos.org/plosone/s/file?id=wjVg/PLOSOne_formatting_sample_main_body.pdf**](https://journals.plos.org/plosone/s/file?id=wjVg/PLOSOne_formatting_sample_main_body.pdf) **and**

[**https://journals.plos.org/plosone/s/file?id=ba62/PLOSOne_formatting_sample_title_authors_affiliations.pdf**](https://track.editorialmanager.com/CL0/https:%2F%2Fjournals.plos.org%2Fplosone%2Fs%2Ffile%3Fid=ba62%2FPLOSOne_formatting_sample_title_authors_affiliations.pdf/1/010f019a31877523-cc2f3198-8e57-4d94-aecf-c1861075ee35-000000/o8kh3xtleoIPts6NVpvAge-ZqjirAqWv9M6QFuA5xOQ=235)**.**

**Authors’ response:** Thank you for pointing out. We carefully checked the requirements and made the necessary amendments (headings, spacing, equation, tables citation and captions). We also changed the first page (title, authors, affiliations) according to the formatting guideline. The changes that corresponded to style adjustments were not marked.

**2. Thank you for stating in your Funding Statement:**

**“This work was supported by the National Council for Scientific and Technological Development, Brazil (CNPq) [28/2018 FOCA Trial 407252/2018-5]. Sacco is a fellow level 1B of the National Council for Scientific and Technological Development (CNPq), Brazil (Process: 302558/2022-5) and Docko holded a scholarship from CNPq (PIBIC). The funders do not have any role in the study and do not have any authority over any study activity or in the decision to submit the report for publication.”**

**Please provide an amended statement that declares *all* the funding or sources of support (whether external or internal to your organization) received during this study, as detailed online in our guide for authors at** [**http://journals.plos.org/plosone/s/submit-now**](http://journals.plos.org/plosone/s/submit-now)**.  Please also include the statement “There was no additional external funding received for this study.” in your updated Funding Statement.**

**Please include your amended Funding Statement within your cover letter. We will change the online submission form on your behalf.**

**Authors’ response:** The funding statement was amended according to the recommendation:

“This work was supported by the National Council for Scientific and Technological Development, Brazil (CNPq) [28/2018 FOCA Trial 407252/2018-5]. Sacco is a fellow level 1B of the National Council for Scientific and Technological Development (CNPq), Brazil (Process: 302558/2022-5) and Docko holded a scholarship from CNPq (PIBIC). All funders did not have any role in the study and do not have any role in the study and do not have any authority over any study activity or in the decision to submit the report for publication. There was no additional external funding received for this study.”

**3. Thank you for uploading your study's underlying data set. Unfortunately, the repository you have noted in your Data Availability statement does not qualify as an acceptable data repository according to PLOS's standards.**

**At this time, please upload the minimal data set necessary to replicate your study's findings to a stable, public repository (such as figshare or Dryad) and provide us with the relevant URLs, DOIs, or accession numbers that may be used to access these data. For a list of recommended repositories and additional information on PLOS standards for data deposition, please see** [**https://journals.plos.org/plosone/s/recommended-repositories**](https://track.editorialmanager.com/CL0/https:%2F%2Fjournals.plos.org%2Fplosone%2Fs%2Frecommended-repositories/1/010f019a31877523-cc2f3198-8e57-4d94-aecf-c1861075ee35-000000/RNk5toBjC8iJxlarwXZUn1eMrYm_B6e2UfGUO1FGCUY=235)**.**

**Authors’ response:** The data set was uploaded at figshare as requested. The datasets generated and/or analyzed during the current study are accessible as anonymized data at <https://doi.org/10.6084/m9.figshare.30546620>.

**4. Thank you for stating the following in the Acknowledgments Section of your manuscript:**

**“This work was supported by the National Council for Scientific and Technological Development, Brazil (CNPq) [28/2018 FOCA Trial 407252/2018-5]. Sacco is a fellow level 1B of the National Council for Scientific and Technological Development (CNPq), Brazil (Process: 302558/2022-5) and Docko holded a scholarship from CNPq (PIBIC). The funders do not have any role in the study and do not have any authority over any study activity or in the decision to submit the report for publication.”**

**We note that you have provided additional information within the Acknowledgements Section that is not currently declared in your Funding Statement. Please note that funding information should not appear in the Acknowledgments section or other areas of your manuscript. We will only publish funding information present in the Funding Statement section of the online submission form.**

**Please remove any funding-related text from the manuscript and let us know how you would like to update your Funding Statement. Currently, your Funding Statement reads as follows:**

**“This work was supported by the National Council for Scientific and Technological Development, Brazil (CNPq) [28/2018 FOCA Trial 407252/2018-5]. Sacco is a fellow level 1B of the National Council for Scientific and Technological Development (CNPq), Brazil (Process: 302558/2022-5) and Docko holded a scholarship from CNPq (PIBIC). The funders do not have any role in the study and do not have any authority over any study activity or in the decision to submit the report for publication.”**

**Please include your amended statements within your cover letter; we will change the online submission form on your behalf.**

**Authors’ response:** The funding statement was amended as “This work was supported by the National Council for Scientific and Technological Development, Brazil (CNPq) [28/2018 FOCA Trial 407252/2018-5]. Sacco is a fellow level 1B of the National Council for Scientific and Technological Development (CNPq), Brazil (Process: 302558/2022-5) and Docko holded a scholarship from CNPq (PIBIC). All funders did not have any role in the study and do not have any role in the study and do not have any authority over any study activity or in the decision to submit the report for publication. There was no additional external funding received for this study.”

The acknowledgment section was also updated as follows:

“The authors acknowledge the Associação Nacional de Assistência ao Diabético (ANAD) for providing the subjects for this research.”

**5. If the reviewer comments include a recommendation to cite specific previously published works, please review and evaluate these publications to determine whether they are relevant and should be cited. There is no requirement to cite these works unless the editor has indicated otherwise.**

**Authors’ response:** Thank you for pointing out, but no recommendations were made by the reviewers.

**6. Please review your reference list to ensure that it is complete and correct. If you have cited papers that have been retracted, please include the rationale for doing so in the manuscript text, or remove these references and replace them with relevant current references. Any changes to the reference list should be mentioned in the rebuttal letter that accompanies your revised manuscript. If you need to cite a retracted article, indicate the article’s retracted status in the References list and also include a citation and full reference for the retraction notice.**

**Authors’ response:** The reference list was reviewed, and no changes were necessary. **Additional Editor Comments (if provided):**

**Please address all the comments made by both the reviewers. Please make sure to review and abide to all journal’s formatting and specific requirements.**

**Authors’ response:** The reviewers’ comments are addressed below on a point-by point basis.

**Reviewers' comments:**

**Reviewer #1:** The authors used a logistic model to determine the interplay between diabetes related risks with risks of fall in a community dwelling adults with DPN. They used a robust methodology to develop and test/validate the model. The sample size is reasonable given the number of predictors they tested. The study is meticulously thought of, designed and the manuscript very well written. Despite these strengths, the title and main aim are somehow NOT CLEAR. While the authors developed and validated the model, the title sounds as if they were merely determining associations. In explanatory logistic regression models the emphasis is mainly on adjusted ORs and p-values (associations). In this case splitting data into training and testing is not necessary. In prediction models however like what the authors have done, AUC, sensitivity and calibrations of the model are the main emphasis. It would be great if the authors consider revising their title and main aim (also enriching their discussion) to reflect exactly what they have done.

**Authors’ response:** Thank you very much for your kind consideration and suggestions. We made some adjustments according to the reviewer’s points:

Title – “Predictive effects of diabetes-related risk factors for falls in community-dwelling people with diabetic peripheral neuropathy based on a logistic regression model”

Aim – We believe that the aim in the manuscript is already bringing emphasis to the development of a predictive model, but we did a slight change to enhance it, both in the main text as in the abstract:

- Abstract: “This study aimed to identify the predictive effects of different aspects of diabetic peripheral neuropathy (DPN) and other already known risk factors for falls through a comprehensive logistic model within community-dwelling older adults with diabetes and DPN. This paper also provides a model that estimates the probability of a fall occurring in a real-world clinical scenario.”
- Main text: “Thus, the aim of this study was to identify predictive risk factors for falls within community-dwelling older adults with DM and DPN, taking into account the DPN severity degree, and to provide a predictive comprehensive logistic model that allows for the estimation of the probability of a fall occurring in a real-world clinical scenario.”

Discussion – “This study provides a comprehensive logistic model that allows for the estimation of the probability of a fall occurring in a real-world clinical scenario, providing a predictive model for falls in this population.”

Overall, we believe that the discussion is already based in the presentation of a predictive model, as suggested by the reviewer, so we did not make any additional amendments. For instance, in the second paragraph we discuss the performance of all the predictive models presented. Here are some additional examples:

“The main findings showed that self-reported balance problems, DPN symptoms, and age represent the strongest risk for falls in people with DM and DPN. Additionally, DPN severity scored by the fuzzy system was also considered a predictive risk factor, suggesting that the combination of loss of sensitivity and DPN symptoms can be associated with an increased risk of falls in persons with DPN.

“Aging has already been cited as a risk factor for falls, even for individuals without DM, due to overall frailty and the presence of comorbidities (James et al., 2020). Its presence in our model supports this evidence. What the current predictive model adds to the understanding of the risk of falls is an estimate of how much this risk increases in the presence of DPN symptoms and/or balance problems.”

“Although Model 2a showed slightly lower performance than Model 1, it indicated that DPN severity is also an important predictive variable for risk of falling in people with DM…”

“In addition, this is the first study to employ a modeling analysis to develop a predictive tool for falls in individuals with DM and DPN using clinical variables from commonly used instruments by healthcare professionals.”

“Finally, this study shows the possibility of predicting the risk for recurrent falls using a model based on clinical variables commonly assessed by healthcare professionals in a real-world setting.”

**Reviewer #2:**

This paper is well written and scientifically sound, with the method section well elaborated, ensuring reproducibility. The scientific findings are backed up and supported with relevant literature. However, the authors are advised to follow the journal instruction on formatting and referencing styles.

**Authors’ response:** Thank you very much for this comment, we are pleased that we met the reviewer’s expectations. The formatting and reference styles were checked and revised, as requested.
